# Peripheral nerve injury sensitizes neonatal dorsal horn neurons to tumor necrosis factor-α

**DOI:** 10.1186/1744-8069-5-10

**Published:** 2009-03-02

**Authors:** Jie Li, Wenrui Xie, Jun-Ming Zhang, Mark L Baccei

**Affiliations:** 1 Pain Research Center, Department of Anesthesiology, University of Cincinnati Medical Center, 231 Albert Sabin Way, Cincinnati, OH 45267, USA

## Abstract

**Background:**

Little is known about whether peripheral nerve injury during the early postnatal period modulates synaptic efficacy in the immature superficial dorsal horn (SDH) of the spinal cord, or whether the neonatal SDH network is sensitive to the proinflammatory cytokine TNFα under neuropathic conditions. Thus we examined the effects of TNFα on synaptic transmission and intrinsic membrane excitability in developing rat SDH neurons in the absence or presence of sciatic nerve damage.

**Results:**

The spared nerve injury (SNI) model of peripheral neuropathy at postnatal day (P)6 failed to significantly alter miniature excitatory (mEPSCs) or inhibitory (mIPSCs) postsynaptic currents in SDH neurons at P9-11. However, SNI did alter the sensitivity of excitatory synapses in the immature SDH to TNFα. While TNFα failed to influence mEPSCs or mIPSCs in slices from sham-operated controls, it significantly increased mEPSC frequency and amplitude following SNI without modulating synaptic inhibition onto the same neurons. This was accompanied by a significant decrease in the paired-pulse ratio of evoked EPSCs, suggesting TNFα increases the probability of glutamate release in the SDH under neuropathic conditions. Similarly, while SNI alone did not alter action potential (AP) threshold or rheobase in SDH neurons at this age, TNFα significantly decreased AP threshold and rheobase in the SNI group but not in sham-operated littermates. However, unlike the adult, the expression of TNFα in the immature dorsal horn was not significantly elevated during the first week following the SNI.

**Conclusion:**

Developing SDH neurons become susceptible to regulation by TNFα following peripheral nerve injury in the neonate. This may include both a greater efficacy of glutamatergic synapses as well as an increase in the intrinsic excitability of immature dorsal horn neurons. However, neonatal sciatic nerve damage alone did not significantly modulate synaptic transmission or neuronal excitability in the SDH, which could reflect a relatively weak expression of TNFα in the injured spinal cord at early ages. The above data suggest that although the sensitivity of the SDH network to proinflammatory cytokines after nerve injury is present from the first days of life, the profile of spinal cytokine expression under neuropathic conditions may be highly age-dependent.

## Background

Peripheral nerve injury in the adult evokes significant changes in both excitatory and inhibitory synaptic signaling within the superficial dorsal horn (SDH) of the spinal cord which result in central sensitization and subsequent hypersensitivity to pain [1-5]. However, it is still unknown if alterations in synaptic efficacy occur in the immature dorsal horn following nerve damage during the early postnatal period. Recent behavioral studies suggest that the SDH network may respond to nerve injury in an age-dependent manner, as the spared nerve injury (SNI) or chronic constriction injury (CCI) models of neuropathic pain fail to evoke persistent mechanical allodynia in rats if the injury occurs at a young age [6]. This may partly reflect a weaker microglial response in the neonatal spinal cord after nerve injury compared to the adult [7,8], given the well-documented role of spinal microglia in the generation of neuropathic pain [9]. Alternatively, it is possible that immature dorsal horn neurons are unresponsive to the factors released from activated microglia (or astrocytes), such as proinflammatory cytokines, which are known to increase the excitability of adult dorsal horn neurons under neuropathic conditions. To date no studies have examined the sensitivity of immature dorsal horn neurons to cytokines following peripheral nerve damage.

Mounting evidence suggests that the proinflammatory cytokine tumor necrosis factor-alpha (TNFα) is an important contributor to pain hypersensitivity following nerve damage in the adult, as interference with TNFα signaling attenuates hyperalgesia and mechanical allodynia after CCI [10], spinal nerve ligation [11] and ventral root transection (VRT) [12]. Nerve injury increases the expression of TNFα in DRG neurons [13,14] as well as in astrocytes, microglia and neurons within the adult SDH [12]. In addition, the TNFα receptor TNFR1 is upregulated in both adult DRG and dorsal horn neurons after VRT [12], which may explain the observation that the spinal application of TNFα produces long-term potentiation (LTP) of C-fiber evoked field potentials in the dorsal horn of injured, but not intact, rats [15]. It seems likely that the observed effects of TNFα after nerve injury at least partially reflect alterations in synaptic function within the adult SDH, as TNFα enhances the frequency of miniature excitatory postsynaptic currents (mEPSCs) and increases AMPA or NMDA-induced currents in naïve SDH neurons [16,17]. Nonetheless, the cellular mechanisms underlying the emergent sensitivity to TNFα after nerve injury are not fully understood and could involve changes in the intrinsic membrane excitability of SDH neurons in addition to the modulation of their synaptic inputs.

The present study was therefore undertaken to characterize the effect of neonatal sciatic nerve injury on the excitability of immature SDH neurons and their sensitivity to TNFα. The results demonstrate that while peripheral nerve injury fails to significantly modulate spontaneous excitatory neurotransmission or neuronal excitability in developing SDH neurons, these same properties become susceptible to regulation by TNFα under neuropathic conditions.

## Methods

All experiments adhered to animal welfare guidelines established by the University of Cincinnati Institutional Animal Care and Use Committee.

### Spared Nerve Injury (SNI)

Sprague Dawley rats (postnatal day 6 for neonates, 200–220 g for adults) were anesthetized with isoflurane and placed on a heating pad maintained at 37°C. The sciatic nerve was exposed at the mid-thigh level, and the common peroneal and tibial branches were ligated with 6-0 suture and transected, leaving the sural nerve intact [18]. Sham operations, in which the sciatic nerve was exposed but not damaged, were used as controls.

### Preparation of spinal cord slices

Pups (P9-P11) were deeply anesthetized with sodium pentobarbital (30 mg/kg) and then perfused transcardially with ice-cold dissection solution consisting of (in mM): 250 sucrose, 2.5 KCl, 25 NaHCO_3_, 1.0 NaH_2_PO_4_, 6 MgCl_2_, 0.5 CaCl_2_, and 25 glucose continuously bubbled with 95% O_2_/5% CO_2_. The lumbar spinal cord was isolated and immersed in low-melting-point agarose (3% in above solution; Invitrogen, Carlsbad, CA) and parasagittal slices (350–400 μm) were cut from the ipsilateral side using a Vibroslice tissue slicer (HA-752; Campden Instruments, Lafayette, IN). The slices were placed in a chamber filled with oxygenated dissection solution for 30 min then incubated for a minimum of 2 h at room temperature in either: *(1) *oxygenated artificial cerebrospinal fluid (aCSF) containing (in mM):125 NaCl, 2.5 KCl, 25 NaHCO_3_, 1.0 NaH_2_PO_4_, 1.0 MgCl_2_, 2.0 CaCl_2_, and 25 glucose supplemented with tumor necrosis factor-α (1 ng/ml); or *(2) *aCSF plus the equivalent amount of vehicle solution (0.1% BSA in PBS). Subsequent electrophysiological studies (see below) were performed by an experimenter blinded to the incubation conditions.

### Patch clamp recordings

Slices were transferred to a submersion-type recording chamber (RC-22; Warner Instruments, Hamden, CT), mounted on the stage of an upright microscope (BX51WI, Olympus, Center Valley, PA) and perfused at room temperature with oxygenated aCSF at a rate of 1.5–3 ml/min.

Patch electrodes were constructed from thin-walled single-filamented borosilicate glass (1.5 mm outer diameter; World Precision Instruments, Sarasota, FL) using a microelectrode puller. Pipette resistances ranged from 5 to 7 MΩ and seal resistances were > 1 GΩ. For voltage-clamp experiments, patch electrodes were filled with a solution containing the following (in mM): 130 Cs-gluconate, 10 CsCl, 10 HEPES, 11 EGTA, 1.0 CaCl_2_, and 2.0 MgATP, pH 7.2 (300–305 mOsm). Current clamp experiments used an electrode solution of (mM): 130 potassium gluconate, 10 KCl, 10 HEPES, 1.0 EGTA, 0.1 CaCl_2_, 2.0 MgATP, pH 7.2 (300–305 mOsm).

Dorsal horn neurons were visualized with infrared-differential interference contrast and whole-cell patch-clamp recordings were obtained as described previously [19]. Sampled neurons were located within the translucent band apparent in spinal cord slices under low magnification, and were thus judged to reside in lamina II of the dorsal horn. EPSCs were isolated at a holding potential (hp) of -70 mV while IPSCs were recorded at a hp of 0 mV, thus minimizing the contribution of NMDA and AMPA/kainate receptor-mediated events [20]. Miniature postsynaptic currents (mPSCs) were isolated via the bath application of 500 nM TTX. In some experiments, EPSCs were evoked via focal electrical stimulation (0–100 μA, 100 μs duration) delivered via a second patch electrode placed near the cell of interest which was connected to a constant-current stimulator (Master-8, Jerusalem, Israel). To investigate whether TNFα altered the probability of glutamate release in the dorsal horn, pairs of identical stimuli (at 2× threshold at a frequency of 0.10 Hz) were delivered at various interstimulus intervals (50–250 ms; 10 trials each), and the paired-pulse ratio (PPR) was calculated as: PPR = Mean EPSC2/Mean EPSC1. To calculate the ratio of AMPAR/NMDAR currents, EPSCs were evoked from a holding potential of +50 mV at a frequency of 0.10 Hz in the presence of 10 μM gabazine and 0.5 μM strychnine. Upon verification of a stable baseline current amplitude, AP-5 was bath applied at 50 μM to block the NMDAR component of the composite current, and the NMDAR-mediated response was subsequently obtained via electronic subtraction. To evoke action potential discharge in SDH neurons, current was injected through the patch electrode in the current clamp configuration (0–60 pA in 10 pA steps; 1 sec duration).

Membrane voltages were adjusted for liquid junction potentials (approximately-14 mV) calculated using JPCalc software (P. Barry, University of New South Wales, Sydney, Australia; modified for Molecular Devices). Currents were filtered at 4–6 kHz through a -3 dB, four-pole low-pass Bessel filter, digitally sampled at 20 kHz, and stored on a personal computer (ICT, Cincinnati, OH) using a commercially available data acquisition system (Digidata 1440A with pClamp 10.0 software; Molecular Devices).

### Measurement of cytokine levels in the dorsal horn

One, three, or seven days following SNI, rats were anesthetized with sodium pentobarbital and perfused transcardially with dissection solution as described above. The vertebral column was rapidly removed, immersed in dissection solution and the spinal cord ejected via hydraulic extrusion. The spinal cord was dissected into quadrants and the ipsilateral L4/L5 dorsal horn was flash frozen on dry ice and stored at -80°C until use. The concentration of TNFα in the dorsal horn of sham and SNI animals were simultaneously quantified using a multiplex assay kit (MILLIPLEX; Millipore; Billerica, MA) based on Luminex X-Map technology as described previously [21]. Dorsal horn tissue (25 μl) from each sample was run in duplicate. Cytokine concentrations from the nerve-injured groups were normalized to the mean concentration observed in the corresponding sham group at a given age.

### Drugs

Recombinant rat TNFα was purchased from R&D Systems (Minneapolis, MN). Tetrodotoxin, D(-)AP-5, and SR-95531 hydrobromide (gabazine) were purchased from Tocris (Ellisville, MO). Strychnine hydrochloride was obtained from Sigma (St. Louis, MO). All drugs were bath applied at 1.5–3 ml/min.

### Data analysis and statistics

mPSCs were analyzed via visual inspection using Mini Analysis (version 6.0.3; Synaptosoft, Decatur, GA) while evoked EPSCs were analyzed using Clampfit (Molecular Devices) software. The threshold for mPSC detection was set at twice the mean amplitude of the background noise. Two-way ANOVAs (with Bonferroni post-tests) were employed to determine whether SNI or TNFα treatment significantly affected mPSC properties or intrinsic firing properties. Because we have previously observed that mPSC frequencies often fail to exhibit a normal distribution in newborn dorsal horn neurons, in these cases data were normalized via log transformation before the two-way ANOVAs were performed. Two-group comparisons were performed with Mann-Whitney tests unless otherwise stated. Data are expressed as means ± SEM.

## Results

### TNFα selectively potentiates glutamatergic synaptic efficacy in the immature dorsal horn under neuropathic conditions

**Figure 1:**
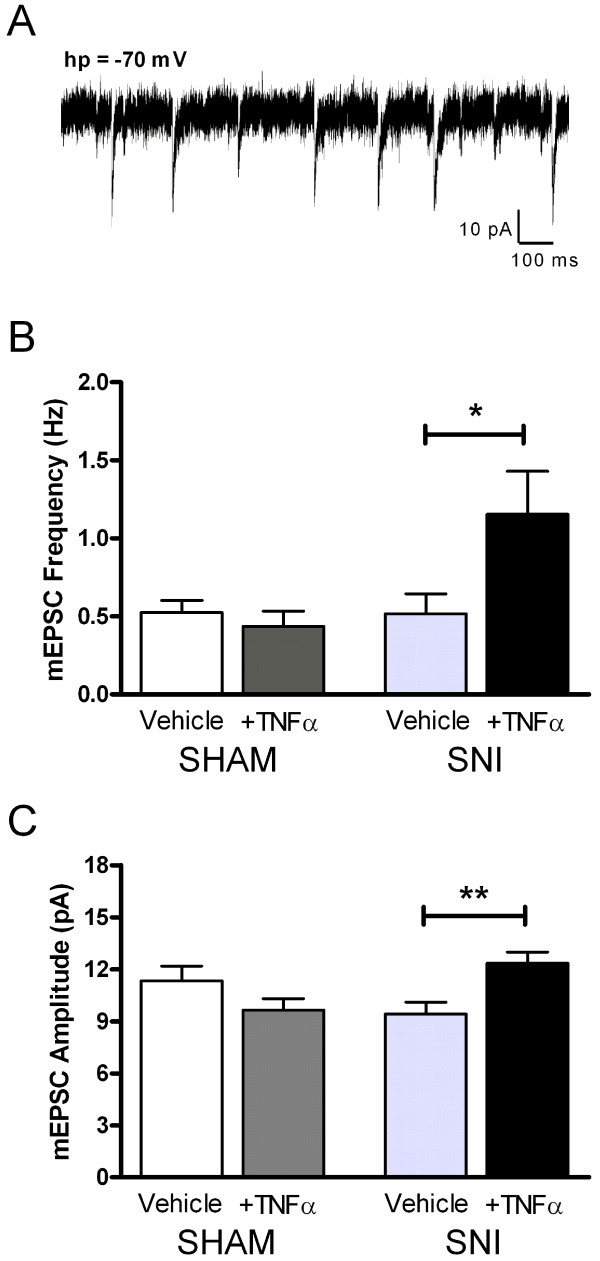
**TNFα incubation strengthens excitatory synaptic efficacy in the immature SDH under neuropathic conditions**. **(A) **Example of mEPSCs observed at a holding potential (hp) of -70 mV in a neonatal SDH neuron. **(B) **Incubation of spinal cord slices with TNFα (1 ng/ml) significantly increased mEPSC frequency in P9-11 SDH neurons after SNI at P6 (*right*; *p < 0.05 compared to SNI+Vehicle; two-way ANOVA) but not in sham-operated littermates *(left)*. **(C) **Similarly, TNFα significantly increased mEPSC amplitude in SDH neurons after SNI (*right*; **p < 0.01 compared to SNI+Vehicle; two-way ANOVA) but not following sham operations *(left)*. The SNI alone did not significantly affect mEPSC frequency **(B) **or amplitude **(C)**.

**Figure 2:**
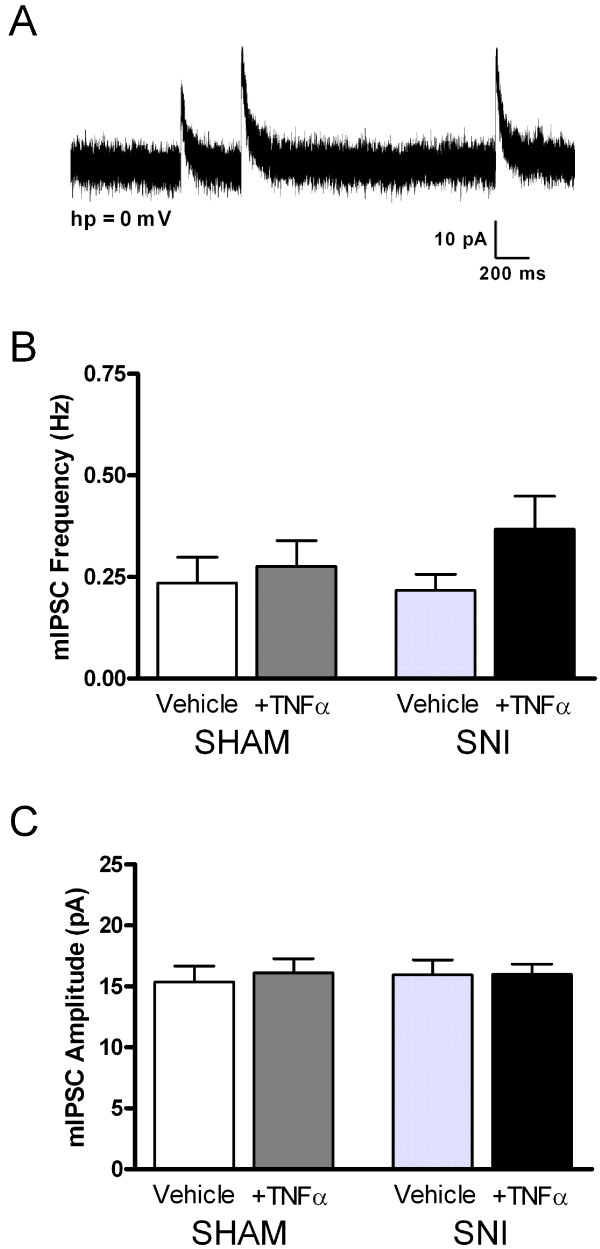
**Spontaneous inhibitory signaling in the neonatal SDH is unaffected by peripheral nerve injury or TNFα incubation**. **(A) **Example of mIPSCs observed at a holding potential (hp) of 0 mV in a neonatal SDH neuron. There were no significant differences in the mean frequency **(B) **or amplitude **(C) **of mIPSCs between any of the experimental groups of P9-11 SDH neurons (p > 0.05; two-way ANOVA) following SNI or sham at P6.

Spontaneous synaptic transmission was characterized in the neonatal superficial dorsal horn (SDH) 3–5 days after peripheral nerve damage occurring at P6. As illustrated in Figure 1, SNI failed to significantly affect the frequency (Sham+Vehicle: 0.53 ± 0.08 Hz, n = 19; SNI+Vehicle: 0.52 ± 0.13 Hz, n = 25; p > 0.05; two-way ANOVA) or amplitude (Sham+Vehicle: 11.33 ± 0.84 pA; SNI+Vehicle: 9.40 ± 0.70 pA) of miniature excitatory postsynaptic currents (mEPSCs) in P9-11 SDH neurons. However, SNI did increase the sensitivity of immature excitatory synapses to TNFα. Following nerve injury, the incubation of spinal cord slices with the cytokine (at 1 ng/ml for ≥ 2 hr) significantly increased both mEPSC frequency (SNI+ TNFα: 1.16 ± 0.27 Hz, n = 23; p < 0.05 compared to SNI+Vehicle; two-way ANOVA; Figure 1B) and mEPSC amplitude (SNI+ TNFα: 12.34 ± 0.64 pA; p < 0.01; Figure 1C), while treating slices taken from sham-operated littermates with the same concentration of TNFα had no effect on glutamatergic signaling (Figure 1B, C). In contrast, we observed no significant effects of either SNI or TNFα on the properties of inhibitory transmission onto the same neurons at this time point (Figure 2), suggesting that TNFα selectively facilitates excitatory signaling in the immature dorsal horn following peripheral nerve injury.

**Figure 3:**
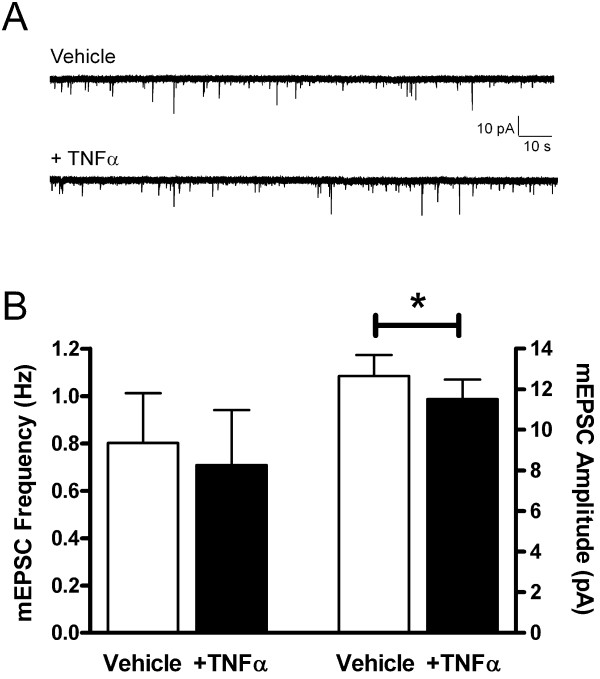
**Acute application of TNFα fails to strengthen excitatory synapses in the neonatal SDH following SNI**. **(A) **Representative traces illustrating mEPSCs in a P10 SDH neuron before *(top) *and 5 minutes after *(bottom) *the bath application of TNFα (1 ng/ml). Continued perfusion of the slice with TNFα for a period of up to 10 min failed to facilitate excitatory synaptic transmission in the dorsal horn. **(B) **The acute application of TNFα did not significantly alter the mean frequency *(left) *of mEPSCs recorded in P9-11 SDH neurons following SNI at P6, and produced a significant decrease in mEPSC amplitude (*right*; *p = 0.024; Wilcoxon matched pairs test) in the same neurons, suggesting that prolonged exposure to TNFα is required for the potentiation of glutamatergic synaptic efficacy in the injured SDH.

Recent work in the adult dorsal horn has demonstrated that TNFα rapidly (within minutes) increases the frequency of spontaneous and miniature EPSCs in a subset of lamina II cells in the absence of peripheral nerve injury [16,17]. To determine if TNFα acutely increases excitatory synaptic strength in the immature SDH under neuropathic conditions, we examined the effects of a bath application of TNFα (1 ng/ml for 10 minutes) on mEPSCs recorded in P9-11 SDH neurons from pups which had undergone SNI at P6. The acute application of TNFα failed to increase the efficacy of excitatory synapses in the neonatal SDH (Figure 3), as there was no significant difference in mean mEPSC frequency in the presence of TNFα compared to baseline measurements taken in the same neurons immediately before TNFα perfusion (Vehicle: 0.80 ± 0.21 Hz; TNFα: 0.71 ± 0.23 Hz; n = 19; p = 0.702; Wilcoxon matched pairs test). In fact, we observed a slight but statistically significant decrease in mEPSC amplitude after the bath application of TNFα (Vehicle: 12.65 ± 1.04 pA; TNFα: 11.51 ± 0.96 pA; p = 0.024; Wilcoxon matched pairs test; Figure 3B). Similar results were obtained using a higher concentration of TNFα (10 ng/ml), as no increase in mEPSC frequency (Vehicle: 0.37 ± 0.16 Hz; TNFα: 0.34 ± 0.18 Hz; n = 4; p = 0.625) or mEPSC amplitude (Vehicle: 11.92 ± 1.11 pA; TNFα: 8.29 ± 0.89 pA; p = 0.125) were seen (data not shown). These data strongly suggest that the TNFα-induced facilitation of glutamatergic transmission following peripheral nerve injury (Figure 1) reflects relatively slow modifications at excitatory synapses in the immature SDH.

### TNFα increases the AMPA/NMDA ratio in immature SDH neurons after SNI

**Figure 4:**
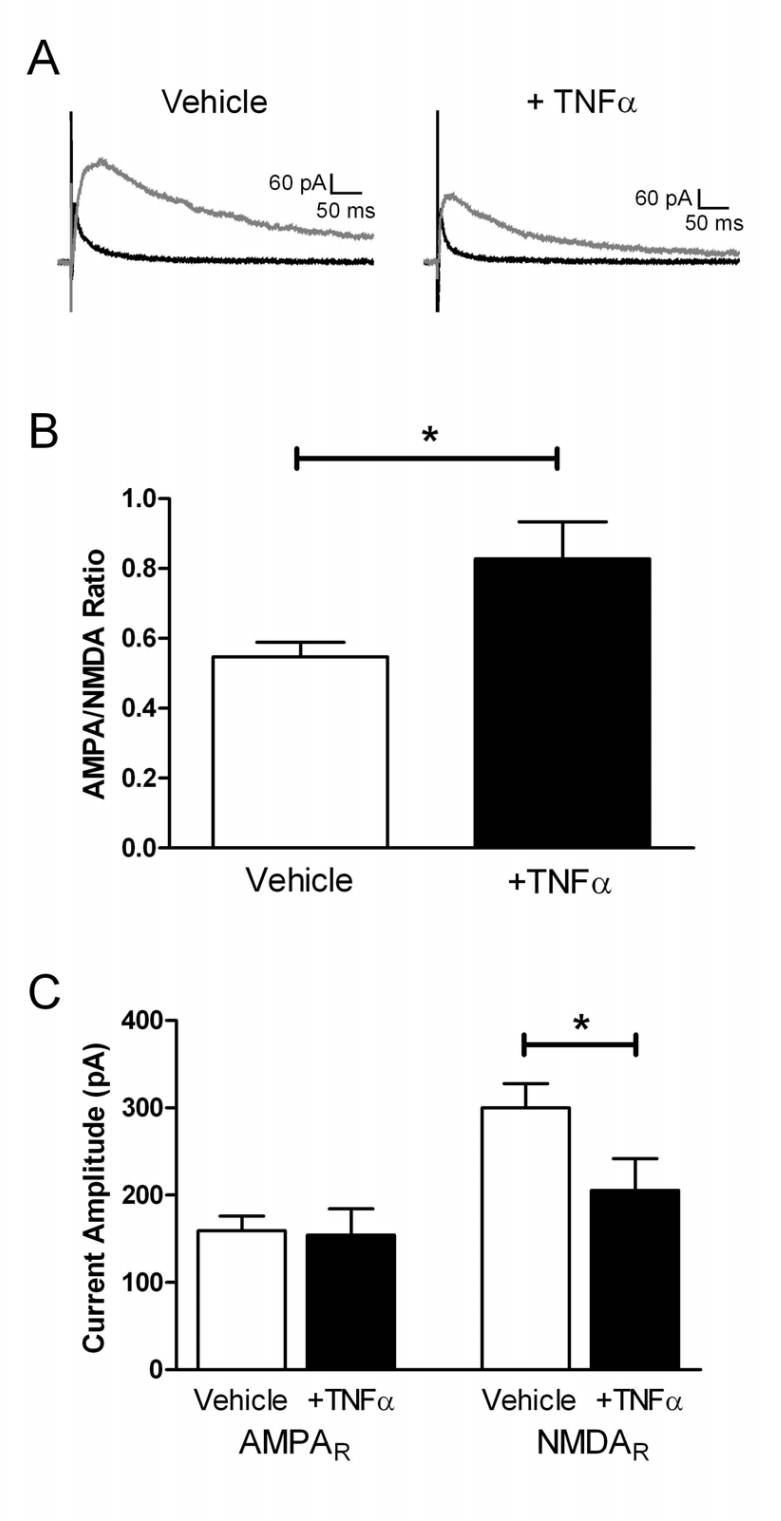
**TNFα reduces NMDAR function in neonatal SDH neurons following nerve injury during early postnatal development**. **(A) **Representative traces of AMPAR-mediated *(black) *and NMDAR-mediated *(gray) *EPSCs recorded from immature SDH neurons (following SNI at P6) after incubation with vehicle solution *(left) *or TNFα *(right)*. Each EPSC represents the average of 10 responses evoked from a holding potential of +50 mV via focal stimulation within the SDH (see Methods). **(B) **TNFα significantly increased the ratio of AMPAR-to-NMDAR current amplitudes compared to vehicle controls (*p = 0.022; Mann-Whitney test). **(C) **Incubation with TNFα did not change the mean amplitude of evoked AMPAR currents *(left) *in SDH neurons under neuropathic conditions, but did significantly decrease NMDAR current amplitudes (*right*; *p = 0.024; Mann-Whitney test).

If the elevation in mEPSC frequency following TNFα incubation (Figure 1) predominantly reflects a postsynaptic effect involving the insertion of AMPARs at new synaptic sites, one might also expect to observe an increase in the AMPAR/NMDAR ratio resulting from a significant increase in the amplitude of evoked AMPAR-mediated EPSCs. To determine if TNFα altered the relative contribution of AMPA and NMDA receptors to synaptic transmission in the neonatal SDH under neuropathic conditions, spinal cord slices were incubated with either TNFα or vehicle solution at P9-11 following SNI at P6 (as described above). Pharmacologically isolated monosynaptic EPSCs were evoked via focal stimulation from a holding potential of +50 mV (see Methods). The AMPAR-mediated component of the evoked response was isolated via the bath application of the selective NMDAR antagonist AP-5 (50 μM) and electronic subtraction subsequently used to obtain the NMDAR-mediated component (Figure 4A). Treatment with TNFα significantly increased the ratio of AMPAR-mediated to NMDAR-mediated current amplitudes compared to incubation with vehicle alone (Vehicle: 0.55 ± 0.04, n = 21; TNFα: 0.83 ± 0.11, n = 16; p = 0.022; Mann-Whitney test; see Figure 4B). Surprisingly, this increase in the AMPA/NMDA ratio reflected a downregulation in NMDAR function (Figure 4A, C), as TNFα significantly decreased the amplitude of NMDAR currents (Vehicle: 300.2 ± 27.7 pA; TNFα: 205.5 ± 36.4 pA; p = 0.024; Mann-Whitney test) while the mean amplitude of AMPAR-mediated responses was similar in the two groups (Vehicle: 159.6 ± 16.7 pA; TNFα: 154.6 ± 29.4 pA; p = 0.635). Overall, these data indicate that the increased mEPSC frequency after TNFα cannot be easily explained by a purely postsynaptic effect involving AMPAR insertion into the postsynaptic membrane, and thus suggest a presynaptic mechanism may be involved.

### TNFα decreases the paired-pulse ratio of evoked EPSCs in immature SDH neurons after SNI

**Figure 5:**
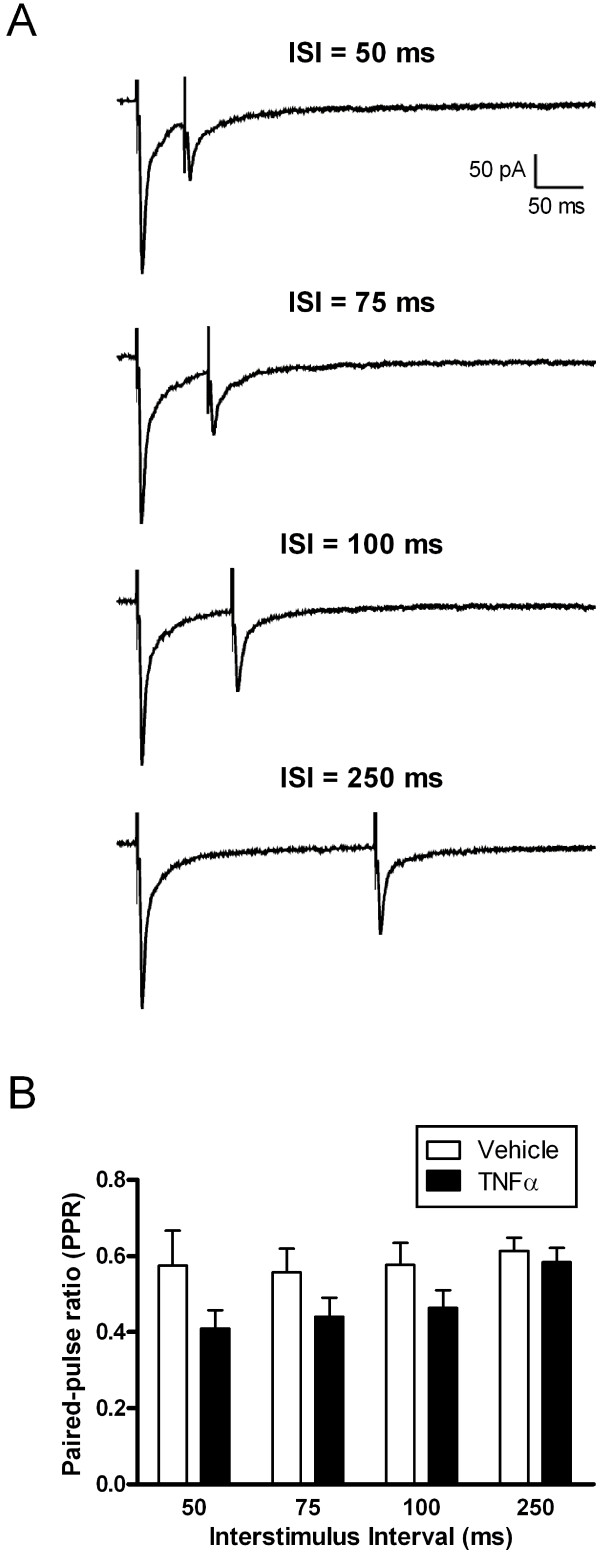
**TNFα decreases the paired-pulse ratio of evoked EPSCs in immature SDH neurons under neuropathic conditions**. **(A) **Representative traces of monosynaptic EPSCs evoked by paired focal stimulation at a variety of interstimulus intervals (ISI) from a holding potential of -70 mV. Each trace represents the average of 10 evoked EPSCs. **(B) **Incubation with TNFα significantly decreased the paired-pulse ratio (PPR) across the range of ISI examined (p = 0.007; two-way ANOVA).

To investigate the possibility that TNFα increases the probability of glutamate release (*P*_*r*_) in the neonatal SDH following nerve injury, spinal cord slices were prepared at P9-11 following SNI at P6 and subsequently incubated with either TNFα (1 ng/ml) or vehicle solution (as described previously). Pairs of electrical stimuli were applied at various interstimulus intervals (ISI; 50–250 ms) using focal stimulation within the SDH (Figure 5A), and the paired-pulse ratios (PPR = Mean EPSC2/Mean EPSC1) of evoked monosynaptic EPSCs were measured. The mean amplitude of EPSC1 was slightly higher in the TNFα group (368 ± 44.4 pA, n = 23) compared to vehicle controls (329 ± 47.8 pA, n = 22) although this difference was not statistically significant (p = 0.208; Mann Whitney test; data not shown). However, as demonstrated in Figure 5B, treatment of slices with TNFα led to a significant decrease in the PPR across the range of ISI examined (Vehicle: n = 20; TNFα: n = 23; p = 0.007; two-way ANOVA). This suggests that TNFα elevates *P*_*r *_at excitatory synapses within the immature SDH under neuropathic conditions, which likely contributes to the observed increase in mEPSC frequency (Figure 1).

### Intrinsic excitability of neonatal SDH neurons is increased by TNFα after SNI

**Figure 6:**
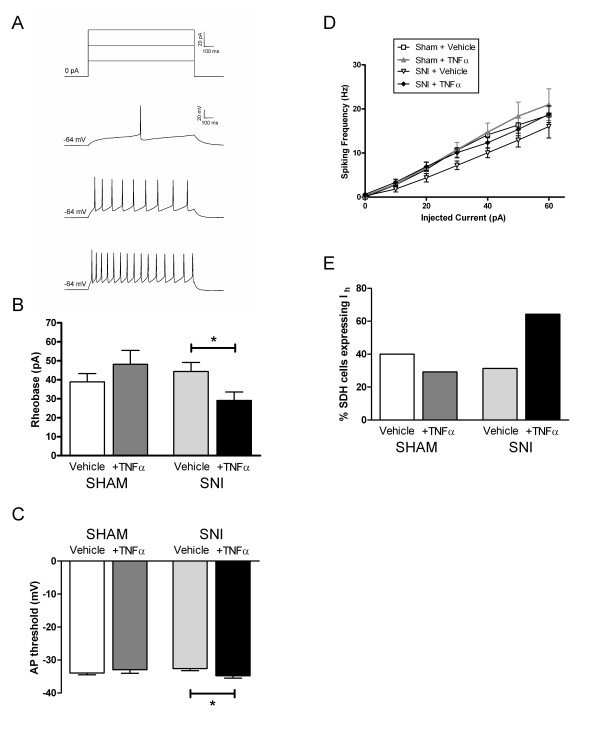
**Intrinsic excitability of immature dorsal horn neurons is increased by TNFα under neuropathic conditions**. **(A) **Example of current-clamp recording from an immature SDH neuron exhibiting a tonic pattern of AP discharge in response to direct current injections of increasing amplitude. **(B) **Incubation with TNFα significantly decreased the current intensity required to evoke AP firing (rheobase) in SDH cells following SNI (*right*; *p < 0.05 compared to SNI+Vehicle; two-way ANOVA) but not in sham-operated littermates *(left)*. SNI alone did not significantly change rheobase levels. **(C) **Similarly, while the SNI itself failed to modulate AP threshold in SDH neurons, TNFα lowered AP threshold in the SNI group (*right*; *p < 0.05 compared to SNI+Vehicle; two-way ANOVA) but not following sham operations *(left)*. **(D) **Plot of neuronal firing frequency as a function of the amount of injected current, demonstrating that there were no significant differences between groups in the mean number of APs discharged during the 1 second current injection. **(E) **TNFα increased the fraction of SDH neurons exhibiting the hyperpolarization-activated inward current (I_h_) after nerve injury (p = 0.029; Chi-square test).

Action potentials (APs) were evoked in P9-11 SDH neurons in response to direct current injections (0 – 60 pA, 1 sec duration) through the recording electrode (Figure 6A). As illustrated in Figure 6B, the minimum current intensity needed to elicit AP discharge (i.e. rheobase) was not significantly altered by SNI at P6 compared to sham-operated littermate controls (Sham+Vehicle: 38.9 ± 4.5 pA, n = 33; SNI+Vehicle: 44.4 ± 4.7 pA, n = 36). However, rheobase levels were significantly decreased by TNFα incubation following peripheral nerve injury (SNI+TNFα: 29.1 ± 4.4 pA, n = 28; p < 0.05 compared to SNI+Vehicle; two-way ANOVA; Figure 6B) but not after the sham operation (Sham+TNFα: 48.1 ± 7.3 pA, n = 24). Similarly, while the AP threshold was not significantly modulated by the SNI itself (Sham+Vehicle: -33.9 ± 0.5 mV; SNI+Vehicle: -32.6 ± 0.6 mV), it was significantly lowered by TNFα following SNI (SNI+TNFα: -34.7 ± 0.5 mV; p < 0.05 compared to SNI+Vehicle; two-way ANOVA; Figure 6C) but not sham surgeries (Sham+TNFα: -32.9 ± 1.1 mV).

To determine whether peripheral nerve injury or TNFα affected repetitive AP discharge in neonatal SDH neurons, we examined the firing frequency during prolonged current injections in a subset of SDH neurons which exhibited a tonic pattern of firing (see Figure 6A). The percentage of sampled SDH neurons firing in a tonic manner ranged from 45–60% and was not significantly affected by SNI or TNFα (p = 0.512; Chi-square test; data not shown). We observed no significant differences in the mean firing frequency between any of the groups (Figure 6D). In addition, there were no significant effects of either SNI or TNFα on AP duration, amplitude, rise rate or decay rate (data not shown). However, following SNI, TNFα did increase the percentage of neonatal SDH neurons that expressed the hyperpolarization-activated inward current (I_h_; p = 0.029; Chi-square test; Figure 6E). Collectively, these results suggest that TNFα causes selective changes in the intrinsic membrane properties of immature SDH neurons which increase neuronal excitability, but these alterations only occur under neuropathic conditions.

### Elevation in spinal TNFα levels following nerve damage depends on postnatal age

**Figure 7:**
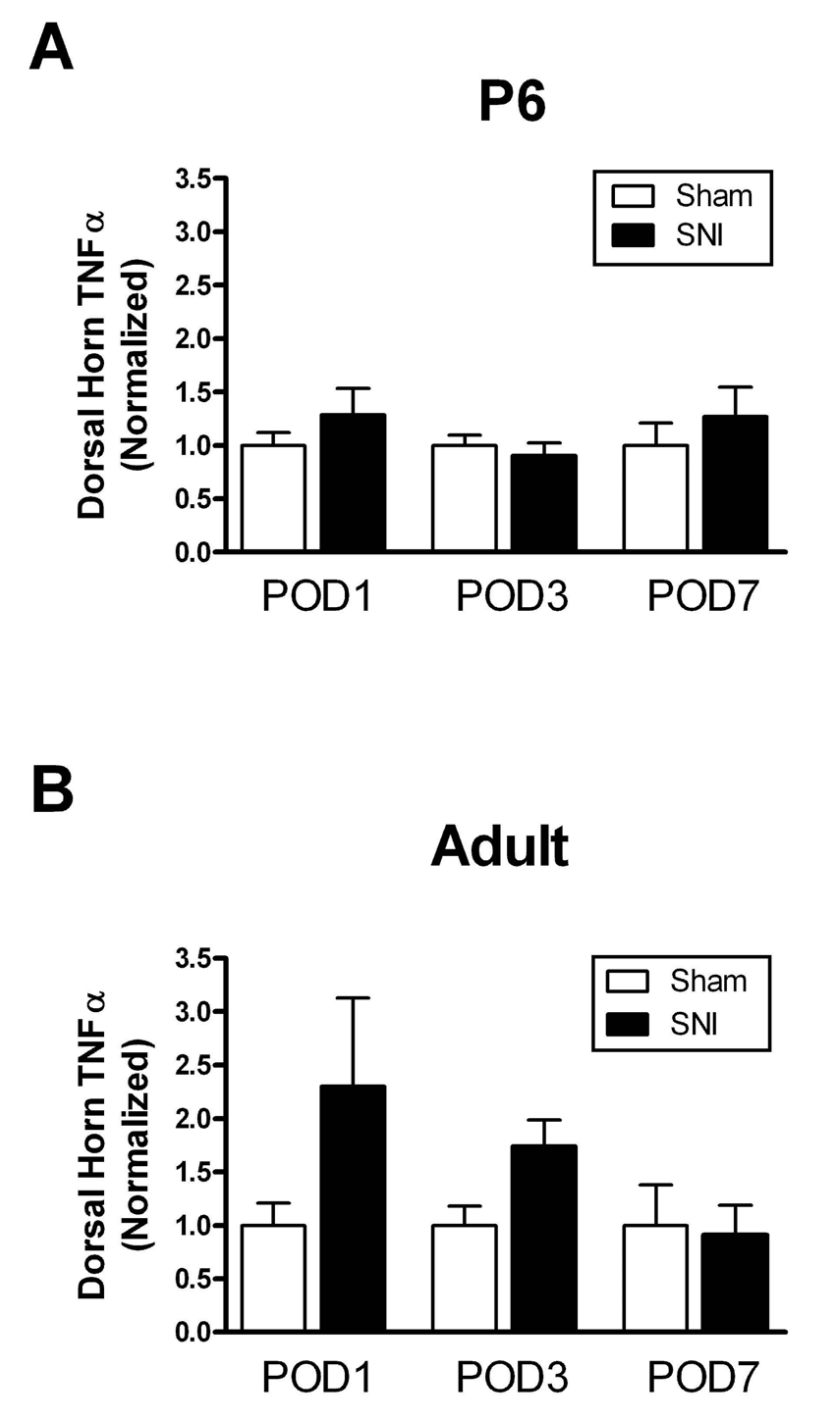
**Peripheral nerve injury elevates TNFα levels in the adult, but not neonatal, dorsal horn**. **(A) **The concentration of TNFα (normalized to sham-operated controls) in the ipsilateral dorsal horn was not significantly altered during the first seven postoperative days (POD) following SNI at P6 (p = 0.276; two-way ANOVA). **(B) **In contrast, adult SNI significantly increased TNFα levels in the ipsilateral dorsal horn over the same postoperative period (*p = 0.045; two-way ANOVA).

The above data demonstrate that TNFα significantly modulates both excitatory synaptic transmission within the neonatal dorsal horn as well as the intrinsic excitability of immature SDH neurons following peripheral nerve injury. However, the data also consistently show that SNI alone during the first postnatal week fails to reproduce the effects of exogenously supplied TNFα. This apparent discrepancy could be explained by an absence of significant TNFα upregulation in the neonatal dorsal horn following peripheral nerve damage. We investigated this issue by measuring the level of TNFα within the ipsilateral lumbar dorsal horn (see Methods) at 1, 3 or 7 days after an SNI administered at either P6 or adulthood (normalized to age-matched sham-operated controls). As illustrated in Figure 7, while SNI significantly increased the expression of TNFα in the adult dorsal horn compared to the sham group during the first postoperative week (n = 4–11 per group; p = 0.045; two-way ANOVA), no such increase was evident in the neonatal dorsal horn following nerve injury at P6 (n = 5–13 per group; p = 0.276; two-way ANOVA). These results suggest that the profile of cytokine upregulation in the dorsal horn following peripheral nerve injury is dependent on postnatal age.

## Discussion

The present data demonstrate for the first time that the immature superficial dorsal horn (SDH) network becomes sensitive to the proinflammatory cytokine TNFα following peripheral nerve injury during the early postnatal period. Exposure to TNFα facilitates excitatory synaptic efficacy in the injured SDH, while inhibitory synapses onto the same neurons are unaffected. In addition, TNFα increases the intrinsic excitability of neonatal SDH neurons under neuropathic conditions. However, despite the observed effects of exogenous TNFα on the injured SDH network, the peripheral nerve injury alone was insufficient to evoke these alterations in glutamatergic synaptic strength or neuronal excitability, which may be explained by the failure of SNI at early ages to significantly elevate spinal levels of TNFα. Collectively, the results suggest that the ability of the SDH network to respond to proinflammatory cytokines is present from the first days of life.

Although the signaling pathways underlying the potentiation of glutamatergic synaptic strength by TNFα under neuropathic conditions have yet to be elucidated, the observations that TNFα increases mEPSC frequency (Figure 1) and decreases the paired-pulse ratio (PPR) of evoked EPSCs (Figure 5) suggest that it enhances the probability of glutamate release (*P*_*r*_) within the immature SDH following nerve damage. Interestingly, TNFα causes a similar decrease in the PPR at excitatory synapses in the anterior cingulate cortex [22]. Previous work suggests that a significant fraction of TNFR1 found in the spinal cord is localized to primary afferent terminals [23], and numerous reports have documented a significant increase in the expression of these receptors in DRG neurons following peripheral nerve damage [12-14]. Thus, one potential explanation for the observed elevation in mEPSC frequency in the SDH is an enhanced probability of glutamate release from sensory afferents in the dorsal horn, resulting from TNFR1 upregulation within injured and/or adjacent uninjured [13] DRG neurons. Since TNFR1 expression is also increased in dorsal horn neurons after nerve injury [12], an upregulation of TNFR1 and subsequent increase in glutamate release probability at the presynaptic terminals of excitatory interneurons may also contribute to the increased mEPSC rate.

It seems surprising that, despite facilitating spontaneous excitatory transmission (Figure 1) and decreasing the PPR of the evoked EPSCs (Figure 5) in neonatal SDH neurons after SNI, TNFα failed to significantly increase the amplitude of evoked AMPAR-mediated EPSCs. However, there are numerous possible explanations for this apparent discrepancy. First, previous studies have demonstrated that agonists which facilitate spontaneous glutamatergic signaling can also reduce the amplitude of electrically evoked EPSCs, possibly by desynchronizing transmitter release at the presynaptic terminals. For example, capsaicin application to neonatal spinal cord slices increases mEPSC frequency in SDH neurons but depresses the evoked EPSCs [24]. In fact, similar effects have recently been reported to occur following the application of TNFα to adult dorsal horn neurons [17]. Second, while the elevation in *P*_*r *_suggested by the PPR experiments (Figure 5) and/or the increase in quantal size (*q*) suggested by the observed elevation in mEPSC amplitude (Figure 1) would certainly predict an increased amplitude of the evoked EPSCs following TNFα (since *i *= n*P_r_*q), this also assumes that one is activating the same number of presynaptic inputs in each experiment (i.e. *n *is constant). The nature of using extracellular focal stimulation in the SDH, a region characterized by enormous heterogeneity, makes it extremely difficult to standardize the number of presynaptic inputs being stimulated across individual cells or different slice preparations. For this reason, a clear advantage of the PPR analysis is that the measurement is independent of the number of stimulated inputs (*n*).

As a result of this caveat, despite the absence of significant changes in the size of evoked AMPAR-mediated EPSCs (Figure 4), we cannot eliminate the possibility that the increased mEPSC frequency following TNFα treatment (Figure 1) also partially reflects an increase in the surface expression of AMPARs within SDH neurons. Previous work has demonstrated that interfering with TNFR1 signaling in hippocampal neurons disrupts AMPAR trafficking to the membrane and decreases both mEPSC frequency and amplitude without altering glutamate release probability [25]. Thus, an increase in TNFR1 expression in SDH neurons under neuropathic conditions could increase the number of functional synapses via AMPAR insertion into the postsynaptic membrane and thus potentiate glutamatergic signaling. However, the decrease in PPR following TNFα treatment (Figure 5) clearly suggests that alterations in the postsynaptic trafficking of AMPARs alone cannot explain the elevation in mEPSC frequency in immature SDH neurons. Interestingly, while TNFR1 activation also decreases the exocytosis of GABA_A_Rs in hippocampal neurons [26], we observed no significant effects of TNFα on inhibitory neurotransmission in the neonatal SDH following peripheral nerve injury (Figure 2).

In any case, the alterations in neonatal SDH synaptic function described here likely involve different mechanisms than those underlying the TNFα-induced potentiation of glutamatergic signaling which was recently described in the adult SDH under naïve conditions [16,17]. First, we observe no significant effect of TNFα on miniature excitatory transmission in the SDH from sham-operated littermates. In addition, while TNFα increased the spontaneous and miniature EPSC frequency in adult SDH neurons within minutes [16,17], neither acute nor prolonged bath application of TNFα (1–10 ng/ml) increased mEPSC frequency or amplitude in neonatal SDH cells following SNI (Figure 3). This suggests that an acute modulation of glutamatergic synapses cannot explain the observed increase in excitatory synaptic strength following TNFα incubation (Figure 1). We thus hypothesize that the TNFα effects result from relatively slow modifications at SDH synapses under neuropathic conditions.

The alteration in AMPA/NMDA ratio produced by TNFα in SDH neurons following SNI (Figure 4B) appears to result from a downregulation in NMDAR function (Figure 4C). Interestingly, a similar decrease in NMDAR function has been previously demonstrated at primary afferent synapses onto young adult lamina I neurons following hindpaw inflammation, which was accompanied by a decrease in the relative contribution of NR2B-containing NMDARs [27]. Thus, the decrease in NMDAR function observed after TNFα treatment could reflect an increased phosphorylation of NR2B subunits in the SDH, which has been previously implicated in both inflammatory and neuropathic pain in the adult [28,29].

Previous studies have demonstrated that TNFα acutely applied to adult rat sciatic nerve *in vivo *increases the firing rate of both A-δ and C-fibers [30], which likely reflects the potentiation of TTX-resistant Na^+ ^currents which occurs in DRG neurons following the activation of TNFR1 and p38 mitogen-activated protein kinase [31]. The sensitivity of sensory neurons to TNFα is enhanced following chronic compression of the DRG (CCD), as TNFα evoked a greater decrease in rheobase in CCD neurons compared to uninjured cells [32]. Less is known about the effects of TNFα on the intrinsic firing properties of dorsal horn neurons. Extracellular single-unit recordings *in vivo *have shown that exogenous TNFα does increase spontaneous action potential (AP) discharge in both wide-dynamic range (WDR) and nociceptive-specific dorsal horn neurons [33], but this technique cannot distinguish between TNFα-induced changes in primary afferent firing, dorsal horn synaptic transmission and alterations in the intrinsic neuronal excitability of dorsal horn cells. To our knowledge, the present data represent the first evidence that this cytokine enhances intrinsic membrane excitability in dorsal horn neurons, as TNFα significantly decreased both rheobase current and AP threshold under neuropathic conditions (Figure 6). These results suggest that the ability of TNFα to induce LTP in the dorsal horn following nerve injury [15] could result from a combination of changes in both SDH synaptic function and the intrinsic membrane properties of SDH neurons.

Pronounced mechanical allodynia, which is a hallmark of peripheral nerve damage in the adult rat, is absent if the injury occurs during the first three postnatal weeks [6]. The present study extends these findings by demonstrating that nerve injury itself fails to modulate synaptic transmission or neuronal excitability in the immature SDH at the time point examined. Although much additional work is needed to fully characterize the effects of cytokines on the function of the developing SDH, our data speculate that the absence of allodynia (and accompanying electrophysiological changes within the SDH) in the neonatal rat is unlikely to be simply explained by an inability of the immature dorsal horn network to respond to proinflammatory cytokines, as TNFα increases the excitability of the injured SDH from the first days of life (Figures 1 and 6). Further evidence arises from recent behavioral work demonstrating that the activation of microglia in the neonatal dorsal horn with intrathecal injections of lipopolysaccharide (LPS) does evoke mechanical allodynia [7], suggesting that the immature SDH can be modulated by immune system activation. Our results also raise the possibility that the absence of allodynia at early postnatal ages could be related to age-dependent differences in the profile of cytokine expression in the dorsal horn, as TNFα levels were significantly increased by SNI in adults but not during the first postnatal week (Figure 7). These results are consistent with previous reports of weaker microglial activation in the neonatal dorsal horn after nerve injury [7], though it should be noted that TNFα expression is not restricted to microglia in the SDH. Overall, the data support the previous hypothesis that specific signals from the injured nerve to the CNS are absent in the immature nervous system [7].

## Conclusion

Our results indicate that peripheral nerve injury at early postnatal ages fails to significantly modulate synaptic efficacy or neuronal excitability in the neonatal rat superficial dorsal horn (SDH), but does sensitize the immature SDH network to the proinflammatory cytokine TNFα. In addition, the data demonstrate age-dependent differences in the spinal expression of TNFα under neuropathic conditions. This study highlights the need for a better understanding of how alterations in neural-immune interactions over the course of postnatal development affect the spinal processing of nociceptive information following peripheral nerve damage.

## List of abbreviations

AP: action potential; AP-5: D-2-amino-5-phosphonovalerate; CCI: chronic constriction injury; CNS: central nervous system; DRG: dorsal root ganglion; LTP: long-term potentiation; mEPSC: miniature excitatory postsynaptic current; mIPSC: miniature inhibitory postsynaptic current; P: postnatal day; SDH: superficial dorsal horn; SNI: spared nerve injury; TNFα: tumor necrosis factor-α; TNFR1: tumor necrosis factor-α receptor 1; TTX: tetrodotoxin; VRT: ventral root transection

## Competing interests

The authors declare that they have no competing interests.

## Authors' contributions

JL performed the surgical procedures, *in vitro *patch clamp recordings, protein measurements, data analysis and statistics, and helped edit the manuscript. WX assisted with the protein measurements and analysis and helped edit the manuscript. JZ assisted with the design of the experiments and editing the manuscript. MB participated in the design and coordination of the experiments and wrote the manuscript. All authors read and approved the final manuscript.
